# Living with parents who smoke predicts levels of toxicant exposure in children

**DOI:** 10.1038/s41598-020-66920-y

**Published:** 2020-07-07

**Authors:** Myung-Bae Park

**Affiliations:** Department of Gerontology Health and Welfare, Pai Chai University, 155-40 Baejae-ro, Seo-gu, Daejeon, 35345 Republic of Korea

**Keywords:** Biomarkers, Diseases, Health care, Medical research, Risk factors

## Abstract

The detrimental effect of secondhand smoke (SHS) on health is well known; due to various factors, efforts to prevent SHS cannot completely eliminate the effect of smoking substances, and SHS has not been sufficiently investigated among children. This study aimed to assess children’s smoke exposure with respect to parents smoking patterns using biomarkers. This study used data from the 2016/2017 Korea National Health and Nutrition Examination Survey. Data pertaining to 486 subjects was extracted. Exposure to smoking among non-smoking children was assessed based on urine levels of 4-(methylnitrosamino)-1-(3-pyridyl)-1-butanol (NNAL). The urine NNAL concentration was highest among children with smoking parents and SHS exposure at home (3.829 pg/mg, 95% confidence interval [CI: 1.499–8.330), followed by children with smoking parents and no SHS exposure at home (1.297, 95% CI: 1.080–1.536), and children with nonsmoking parents and no SHS exposure at home (0.996 pg/mg, 95% CI: 1.026–1.427). Living with a smoking parent was associated with exposure to carcinogens, and a critical predictor of tobacco-specific nitrosamine. Prohibition of smoking at home is effective at preventing SHS in children. However, it cannot completely prevent passive smoking, which might be attributable to thirdhand smoking and undetected secondhand smoke.

## Introduction

A significant body of evidence has shown that cigarettes have a detrimental effect on health of the smoker as well as people who passively inhale smoke^1^. Secondhand smoke (SHS) has been associated with various diseases as well as premature death^1^. SHS is known to be as bad as direct smoking and contains 70 carcinogens such as benzene and Benzopyrene^2,3^, as well as nicotine, which is known to be harmful to health and associated with cancer^4^. For this reason, the International Agency for Research on Cancer (IARC) recognizes SHS as a carcinogen^5^ and campaigns for increased awareness and prevention of the risks of SHS^1^. Children are particularly vulnerable to SHS. This can have an adverse effect on all aspects of their health and development, leading to increase in risk of respiratory diseases and coronary heart diseases as well as poor growth and, in some cases, sudden infant death syndrome^1,6–8^. In addition, exposure to SHS in childhood has been shown to shorten adult lifespan^9^. Children require urgent protection from SHS, especially within their homes^10^. Although SHS exposure at home is declining worldwide, estimates suggest that more than 40% of children are still exposed to SHS at home and other environments they frequently inhabit; the rate of exposure exceeds 50% in the Western Pacific region and reaches 80% in Europe^11^.

However, as the perils of SHS have been scientifically supported and become widely known among the public over the past several decades, understanding of the risks associated with SHS has markedly improved. In the United States, SHS exposure has been significantly reduced during 1988–2014^12^. Furthermore, the percentage of households that consider themselves smoke-free is on the rise, with an increasing number of households enforcing a smoke-free rule^13^. In South Korea, SHS exposure at home among the youth has declined from 40.3% in 2006, when a national-level survey was first launched, to 23.0% in 2018. Understanding the perils of SHS has resulted in lower SHS exposure as parents limited smoking in front of their children, thus, limiting their SHS exposure^13^. Although this rate of decline is noteworthy, the remaining 23% is equivalent to 1 in 5 children still exposed to SHS at home^14^. According to Winickoff *et al*.^15^, most people believe that SHS is bad for children’s health. In addition, in South Korea, 98% of the respondents to a national survey also declared that SHS exposure was detrimental to children’s health, while 94% reported to have banned smoking at home^16^. Effort to prevent children’s SHS exposure at home has been visibly enhanced.

Recently, evidence has shown that efforts to avoid exposure to SHS do not provide sufficient protection against smoking substances. Parents do not smoke in front of children, but smoking from a balcony or near a window in the absence of children can cause smoke to enter the room or spread in the vicinity, causing SHS^17^. Therefore, efforts to prevent SHS may be insufficient to prevent exposure to SHS or thirdhand smoke (THS). THS refers to the smoking substances that remain in carpets, walls in the home, and get re-released into the air. Thus, children may still be exposed to toxic substances from smoking^13,18^.

Studies have been conducted to identify SHS and THS in children at the population level, but the causes of exposure outside the home have not been controlled^19^; and it is not easy to generalize because smoking habits differ depending on the country and culture^20,21^. To the best of our knowledge, this has not been studied in Asian children. Therefore, this study aims to ensure that the efforts to limit smoking in the home to prevent SHS in their homes are protecting their children well with smoking substances. For this, specifically, I examined children’s smoke exposure by measuring biomarkers in: (i) children who live with non-smoking parents and have not been exposed to SHS at home, (ii) children who live with at least one smoking parent and have not been exposed to SHS at home, and (iii) children who live with at least one smoking parent and have been exposed to SHS.

## Material and Methods

### Data sources

We used data from the Korea National Health and Nutrition Examination Survey (KNHANEs). This survey has been conducted by the Korea Centers for Disease Control and Prevention (KCDC) since 2008. KNHANEs survey about 10,000 people aged 1 year or older every year. It uses a multi-stratified cluster sampling method with regional and household-level extraction units, which makes the sample representative of the Korean population. KNHANEs can be divided into three categories: lifestyle survey that covers smoking, drinking, physical activity, subjective health, etc.; physical check-up that includes x-ray, blood, and urine test; and nutritional survey on dietary habit. A lifestyle survey and physical check-up are carried out by a mobile clinic dispatched to the survey target area. Questions related to smoking are asked during a face-to-face interview with children aged 6 years and above. In principle, the data are self-reported answers, however, among respondent children aged 6–18 years, young children under 12 years could respond with the help of their parents. A current smoker is defined as ‘a person who has smoked more than 100 cigarettes in his or her lifetime and who currently smokes cigarettes’. Urine is collected at the time of the physical check-up, whereby the participants are provided with a cylinder in which to collect the sample. The urine samples are then refrigerated and moved to a storage place on the day of collection, where they are kept in a deep freezer with cryogenic freezing at a temperature below −70 °C. On average, a week after the lifestyle survey and physical check-up, the surveyor visits the participant’s home and conducts the nutritional survey.

### Participant selection, exposure definitions, and outcome measures

To quantify smoking exposure among non-smoking children, NNAL (4-(methylnitrosamino)-1-(3-pyridyl)-1-butanol) was selected as the outcome variable. NNAL is a carcinogen produced from smoking^22^. It is used as a biomarker for SHS, as it is a metabolite of nicotine-derived nitrosamine ketotone (NNK), and is a tobacco-specific nitrosamine (TSNA)^23^. TSNA is produced when nicotine and other tobacco alkaloids combust. Whereas cotinine is a biomarker with a half-life of 3–7 days^24^, NNAL has a half-life of 10–21 days, which makes it suitable to assess smoking or SHS within a month of exposure^25^. NNAL in this study was analyzed by HPLC-MS/MS, using Agilent 1200 Series with Triple Quadrupole 5500 (AB Sciex/USA). For validity, internal and external quality control teams carried out the analysis. The results met the criteria of the German External Quality Assessment Scheme for Analyses in Biological Materials (G-EQUAS) and the College of American Pathologists’ (CAP)-Survey. Moreover, the laboratory comparison with the San Francisco General Hospital met the Clinical and Laboratory Standards Institute (CLSI) guidelines. NNAL data has been collected since 2016 and KNHANEs data was now available in 2017. Urinary NNAL is randomly examined in half of all subjects over 6 years of age. This study used data from 2016 and 2017 and included 3,377 non-smoking children aged 6 to 18 years.

**Figure 1:**
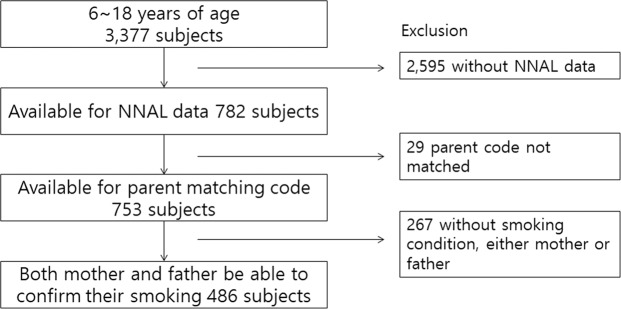
Flow chart of study participants included in this study.

Among them, 2,595 children with missing NNAL data were excluded, and additional 29 children were excluded due to a missing parent code. From the remaining 486 children, 267 were further excluded if smoking status for at least one parent could not be established (Fig. 1). SHS exposure was determined with the answer to the question: ‘Is there anyone at home who has smoked indoor within the last 7 days?’ Answer ‘yes’ defined SHS exposure. To identify SHS exposure in a public place, we asked ‘In the last 7 days, have you been near someone who smoked in a public space?’ To aid responders, we provided examples of public spaces, such as governmental building, school, library, public transportation, hotel and motel, café, restaurant, among others.

### Data analysis

NNAL concentration in children was assessed by dividing the children into: (i) children who live with non-smoking parents, (ii) children who live with at least one smoking parent but no SHS exposure at home, and (iii) children who live with at least one smoking parent and SHS exposure at home. As exposure to smoke varies depending on age and sex, the analysis was stratified by these demographic characteristics^26^. To identify differences in NNAL level between the three groups, ANCOVA and Tukey’s post-hoc tests were performed, adjusting for SHS exposure in public places. The analyses were weighted to reflect the multi-stratified cluster sampling of the survey, and the correlation analysis and ANCOVA used log transformed values.

### Ethics declarations

The data of this study was conducted with the approval of the Institutional Review Board of the Korea Centers for Disease Control and Prevention (2010-02CON-21-C, 2011-02CON-06-C, 2012-01EXP-01-2C, 2013-07CON-03-4C, 2013-12EXP-03-5C, 2018-01-03-PA). Data can be downloaded with permission from the Korean CDC KNHANEs website (https://knhanes.cdc.go.kr/knhanes/eng/).

## Results

There were 251 (53.1%) boys and 235 (46.9%) girls in this study, with a mean age of 12.1 years (95% CI: 11.6–12.5). Furthermore, 301 (52.9%) children were aged 12 years or younger, 104 (23.0%) were aged 13–15 years, and 81 (24.1%) were aged 16–18 years. Among 486 children, 267 (55.1%) lived with nonsmoking parents. In addition, 196 (39.2%) lived with smoking parents but were not exposed to SHS at home, while 23 (5.7%) lived with smoking parents and were exposed to SHS at home. A total of 80 (19.2%) children responded that they have been exposed to SHS in public places in the past week. Exposure to smoke was the highest (61%) among children who lived with smoking parents and have been exposed to SHS at home. The mean number of cigarettes smoked among smoking parents was 14.2 (95% CI: 6.6–21.8); Among parents-smokers whose children were not exposed to SHS at home, the average number of cigarettes smoked was 13.7; for parents whose children were exposed to SHS at home, it was 18 (Table 1).

**Table 1:** Characteristics of parental smoking patterns (number of respondents, weighted percentages).

Respondents (%)		Total	Both non-smoking parents	At least one smoking parent but non-SHS exposure at home	At least one smoking parent and SHS exposure at home
		486 (100.0)	267 (55.1)	196 (39.2)	23 (5.7)
Sex (N = 486)	Boy	251 (53.1)	138 (29.3)	98 (19.9)	15 (3.8)
	Girl	235 (46.9)	129 (25.8)	98 (19.3)	8 (1.9)
Age (N = 486)	Mean	12.1 (11.6–12.5)	12.3 (11.7–12.9)	11.5 (10.8–12.2)	13.7 (12.2–15.2)
	6–12	301 (52.9)	159 (27.4)	133 (23.9)	9 (1.5)
	13–15	104 (23.0)	61 (12.9)	33 (7.3)	10 (2.9)
	16–18	81 (24.1)	47 (14.8)	30 (8.0)	4 (1.3)
SHS exposure at a public place (N = 485)	Non-exposure	405 (80.4)	229 (83.5)	166 (82.1)	10 (39.0)
	Exposure	80 (19.2)	38 (16.5)	29 (17.0)	13 (61.0)
Mean number of cig/day of smoking parents (N = 200)	Overall	14.2 (6.6–21.8)		13.7 (6.1–21.3)	18.0 (11.5–24.4)
	Fathers	15.1 (13.5–16.7)		14.3 (12.7–15.9)	20.1(16.0–24.2)
	Mothers	6.2 (3.7–8.7)		6.4(4.0–8.7)	4.0(–)

All percentages are weighted.

There was a positive correlation between the level of urinary NNAL of fathers, mothers, and children. The urine NNAL concentration in children was positively correlated the urine NNAL concentration in mothers (coeff = 0.2080; *P* = 0.0133) and fathers (coeff = 0.2742; *P* = 0.0007). There was also a positive correlation between the level of urinary NNAL of mothers and fathers (coeff = 0.5045: *P* < 0.0001) (Table 2).

**Table 2:** Correlations between the level of urinary NNAL in children, mothers, and fathers.

N = 486	Children	Mothers	Fathers
Children	1	0.2080 (*P* = 0.0133)	0.2742 (*P* = 0.0007)
Mothers	0.2080 (*P* = 0.0133)	1	0.5045 (*P* < 0.0001)
Fathers	0.2742 (*P* = 0.0007)	0.5045 (*P* < 0.0001)	1

Log-transformed, Creatinine-corrected cotinine.

Children’s urinary NNAL concentrations were examined according to the parents’ smoking characteristics. The average NNAL concentration in the entire children group was 1.217 pg/mg. The NNAL concentration was the highest among children of smoking parents who were also exposed to SHS at home (3.829 pg/mg, 95% confidence interval [CI]: 1.499–8.330), followed by children with smoking parents but no SHS exposure at home (1.297, 95% CI: 1.080–1.536), and children with nonsmoking parents (0.996 pg/mg, 95% CI: 1.026–1.427). Stratifying by sex, boys had a higher concentration of urinary NNAL (1.583 pg/mg, 95% CI: 1.246–1.971) than girls (0.865 pg/mg, 0.748–0.990). Stratifying by age, the NNAL concentration was the highest among the 16–18 years group (1.837 pg/mg, 95% CI: 1.124–2.790), followed by <12 years group (1.071 pg/mg, 95% CI: 0.920–1.234), and 13–15 years group (1.004 pg/mg, 95% CI: 0.723–2.790) (Table 3).

**Table 3:** Children’s urinary NNAL concentration according to parental smoking patterns.

Respondents (%)		Total	Both non-smoking parents	At least one smoking parent but non-SHS exposure at home	At least one smoking parent and SHS exposure at home
(N = 486)	pg/mg (cr)	1.217 (1.026–1.427)	0.996 (0.775–1.244)	1.297 (1.080–1.536)	3.829(1.499–8.330)
Sex (N = 486)	Boy	1.583 (1.246–1.971)	1.272 (0.881–1.744)	1.630 (1.244–2.082)	5.294 (1.713–13.605)
	Girl	0.865 (0.748–0.990)	0.721 (0.606–0.845)	0.997 (0.804–1.210)	1.791 (0.868–3.170)
Age (N = 486)	6–12	1.071 (0.920–1.234)	0.840 (0.719–0.969)	1.271 (1.033–1.536)	3.159 (1.128–7.128)
	13–15	1.004 (0.723–1.330)	0.676 (0.443–0.948)	1.131 (0.795–1.531)	2.794 (1.279–5.317)
	16–18	1.837 (1.124–2.790)	1.700 (0.952–2.736)	1.545 (0.824–2.552)	8.954 (0.348–72.491)

Geometric means and 95% CI.

Creatinine-corrected cotinine.

**Figure 2:**
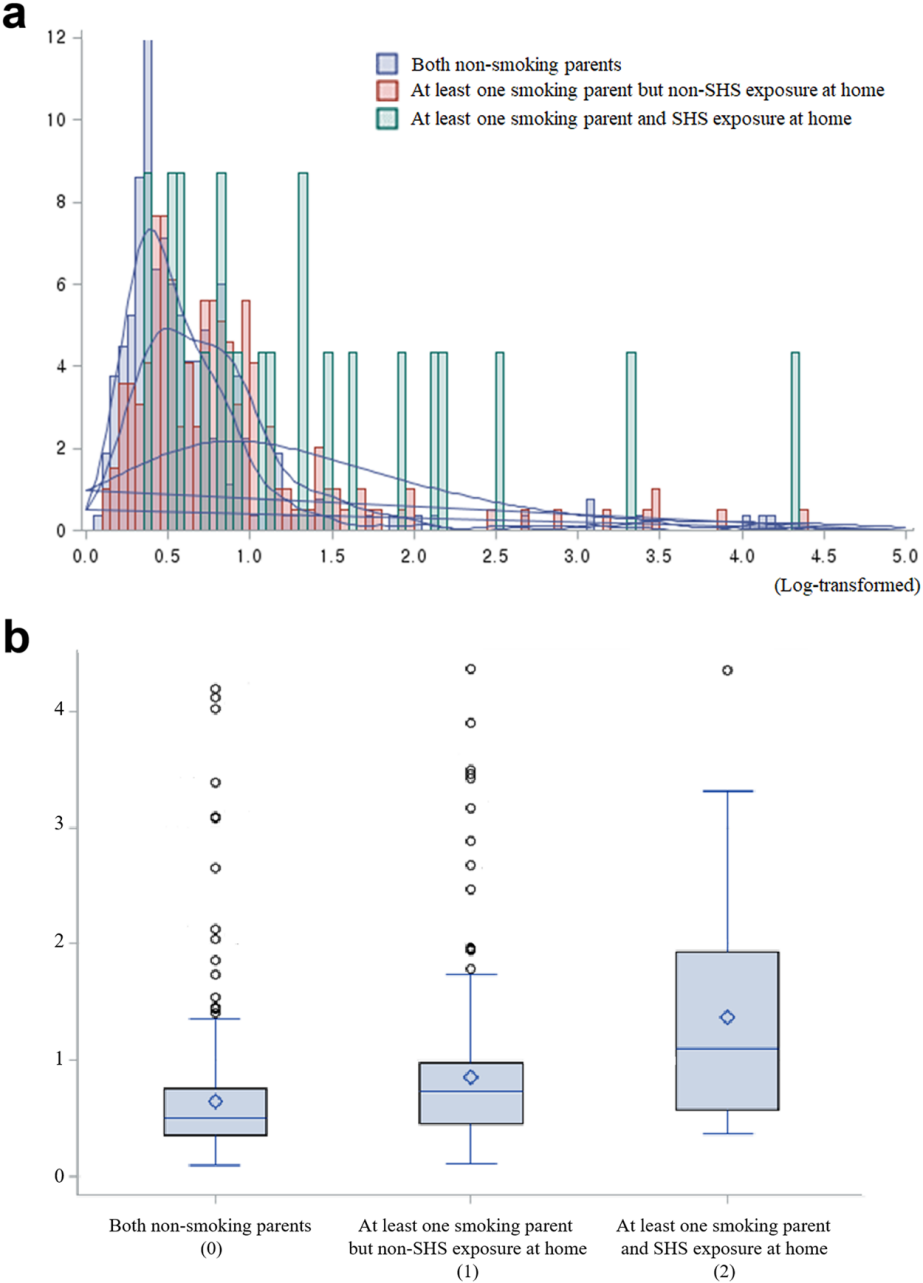
Children’s urinary NNAL level according to parental smoking patterns. (**a**) Children’s urinary NNAL((4-(methylnitrosamino)-1-(3-pyridyl)-1-butanol) distribution according to parental smoking patterns. (**b**) Tukey’s post-hoc test: 0 < 1 < 2 (*p* value of 0.05). Age, sex, and secondhand **smoke** at public places were adjusted.

The results of the ANCOVA and Turkey’s post-hoc tests, using log-transformed data adjusted for age, sex, and SHS exposure at public place, revealed that children with at least one smoking parents and SHS exposure at home had a significantly higher NNAL concentration than children with smoking parents but without SHS exposure at home (diff = 0.694, *P* = 0.022), and children with non-smoking parents and non-SHS exposure at home (diff = 0.860, *P* = 0.007). NNAL in Children with at least one smoking parents but without SHS exposure at home, was significantly higher than NNAL in children with non-smoking parents and non-SHS exposure at home group (diff = 0.166, *P* = 0.019) (Table 4, Fig. 2).

**Table 4:** The results of the ANCOVA analysis with Tukey’s post-hoc tests on the differences in urinary NNAL levels (N = 486, alpha = 0.05).

Parent’s smoking status (A)	Parent’s smoking status (B)	Difference LS means (A)-(B)	P-value	95% Confidence Limits
At least one smoking parent and SHS exposure at home	At least one smoking parent but non-SHS exposure at home	0.694	0.022	(0.101–1.288)
At least one smoking parent and SHS exposure at home	Both non-smoking parents	0.860	0.007	(0.244–1.476)
At least one smoking parent but non-SHS exposure at home	Both non-smoking parents	0.166	0.019	(0.028–0.304)

Age, sex, and SHS exposure in public place were adjusted.

Post-hoc test was used creatinine-corrected cotinine and log-transformed values.

## Discussion

In the present study, 1 in 10 children living with a parent who smokes was exposed to SHS at home. Urine NNAL concentration in these children was on average four times higher than in children of nonsmoking parents, and three times higher than in children with smoking parents but without SHS exposure at home. This indicates that parents’ smoking habit is still a serious threat to the health of many children. In contrast, children who were not exposed to SHS at home had a markedly lower NNAL concentration than children who were exposed to SHS at home. This result is in line with previous findings that have shown that smoke-free rule at home lowers children’s exposure to smoking^27,28^. Nevertheless, enforcing the no-smoking rule at home could not protect smoking parent children’s health. In the present study, we found that children who lived with smoking parents still had a higher level of urine NNAL concentration than children with nonsmoking parents, even if they were not exposed to SHS at home.

This suggests that simply refraining from smoking at home by smoking parents cannot keep children safe from cigarettes. The World Health Organization (WHO) states: “There is no safe level of exposure to tobacco smoke”^1^. Exposure to even a trace amount of substances found in cigarettes can have a detrimental, and sometimes even fatal, effect on health^29^; because even if children are not directly exposed to their parents’ cigarette smoking, cigarette substances attach to parents’ clothes and skin, affecting children indirectly. Alternatively, when people smoke in the absence of children or other family members are not at home, the harmful substances might be adsorbed by the wall or carpet or any other home fixture and then be released into the air^30–32^. When smoking on the balcony, smoke might flow back into the house and get adsorbed on the smoker’s clothes. As a result, some studies suggest that parents’ abstinence from smoking is the only measure that can effectively protect children from exposure to substances contained in cigarettes^13,33^.

In the present study, it is likely that the children of smoking parents without exposure to SHS still had a higher level of NNAL concentration than the children of non-smoking parents due to THS. A previous study has shown that a variety of toxic substances can infiltrate the body through THS^34^. THS poses a significant challenge for the prevention of exposure to smoking. Legislation that created smoke-free spaces combined with individual efforts to prevent SHS is not enough to protect everyone at all times. Multiple studies have warned that THS is equally as damaging as SHS^18,34^,because it emits harmful substances similar to what is emitted through SHS, inducing DNA mutations and damages^35^, each of which is particularly harmful to children^36^. Moreover, there might be SHS of which parents were not aware, because sometimes after smoking, smoking substances are discharged into exhalation. Therefore, children may be exposed to tobacco toxins in parents’ breath when a smoking parent comes in contact with their child after smoking^37^. In other words, efforts by parents who smoke to prevent the exposure of their children to smoke may be insufficient and ineffective due to lack of awareness, many children are still exposed to THS and SHS.

Parents’ smoking is the most important contributor to children’s exposure to SHS and THS, and its effect is particularly detrimental due to the following: First, parents’ smoking may cause substances such as tobacco-specific nitrosamine (TSNA) to be adsorbed by all indoor home surfaces, which then release these substances into the air^34,36^. Second, as children spend a lot of their time at home and in contact with their parents, their frequency of exposure is high. In addition, smoking in public places such as restaurants, and the street might expose people to high concentration of toxic substances intermittently and over a short period. On the other hand, SHS and THS by parents provide continuous exposure over a long period at places of residence^36^. Besides, while SHS is a serious health threat, so is THS especially for children who tend to come into closer contact with surfaces such as rugs and walls where harmful substances from smoke usually linger. In other words, unless smoker parents quit smoking, children remain exposed to carcinogens for prolonged periods. Moreover, even if a parent quits smoking, the residual tobacco exposure is likely to be maintained within the home for a long time.

Although this study controlled SHS in public places, exposure to smoking outside the home may have nevertheless affected NNAL concentrations in children. Parents who smoke at home are less likely to be aware of the risks of children’s exposure to smoking substances^38,39^. When children are with their smoking parents, they can visit the indoor spaces where smoking is allowed more often, and parent smoking outside the home, might have affected them. In addition, the children of smoker parents often encounter tobacco and tend to become familiar with it^40,41^. For this reason, it is possible that these children do not escape from smoking outside the home and have exposed themselves to environments where smoking occurs. In this study, children of parents who smoked at home were also more exposed to SHS in public places. Children exposed to SHS in public places had a 1.5 times higher NNAL concentration compared to those who were not exposed, but there was no statistically significant difference when the sex, age, and parental smoking patterns were adjusted (Appendix figure).

**Appendix figure:**
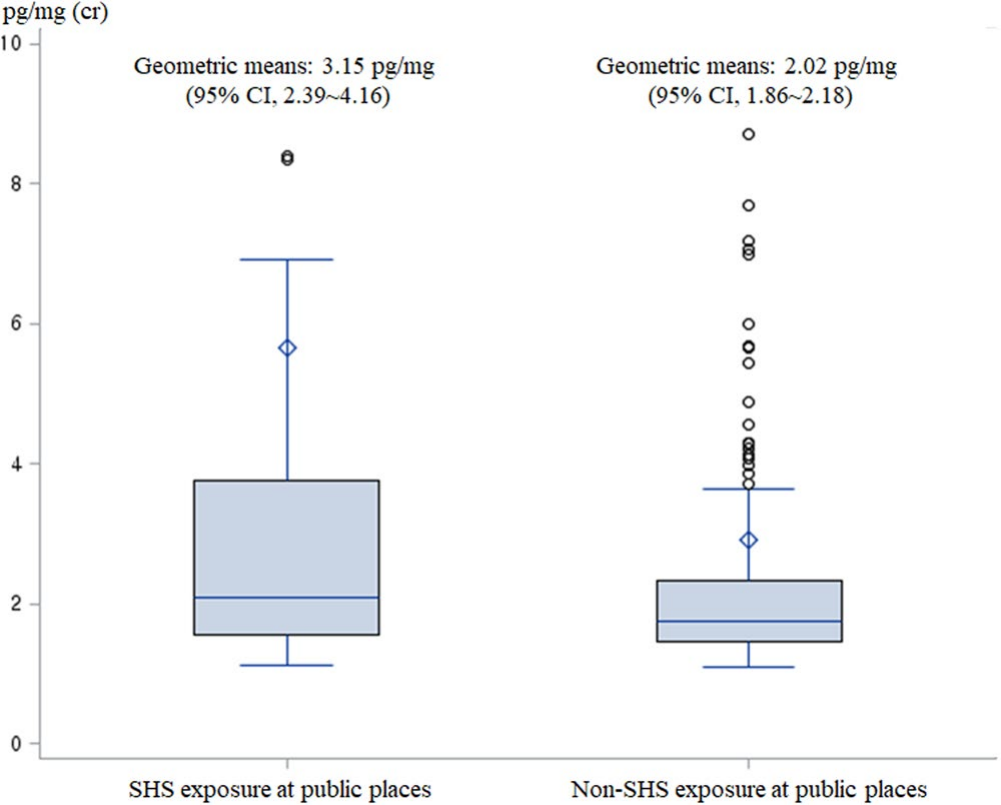
Children’s urinary NNAL concentration according to SHS exposure in public places. And the result of the t-test on the log-transformed data (*T value* = 0.270, *P value* = 0.05). Age, sex, and parental smoking patterns at home were adjusted.

Since SHS is a serious health threat, it had led to smoke-free laws, which countries around the world have implemented^42^. No matter how hard people try to prevent the spread of tobacco smoke, the exposure to smoking substances indoor or at home cannot be completely prevented. Thus, the WHO stated that only ensuring a 100% smoke-free environment indoors can protect the body from tobacco substances^43^. For this reason, several countries continue to reinforce smoke-free zones, and the percentage of places that enforce a complete ban is on the rise^42^. From among the several articles contained in the Framework Convention on Tobacco Control (FCTC), compliance with the “Protection from exposure to tobacco smoke [Article 8]” has achieved the highest rate of adherence^44^ because this is the most obvious way for non-smokers including children to prevent unwanted exposure to smoking, and a wide range of interventions are possible through law and policy^45,46^.

Nevertheless, laws against smoking at home are rare worldwide, and enactment and enforcement of such laws remain difficult for several reasons. As a result, children remain exposed to SHS at home, and even if they are not exposed to SHS at home, and if the parents have health awareness and do not expose their children to SHS, the children remain exposed to THS. Therefore, in addition to implementing relevant policy, strictly prohibiting smoking at home is the best measure to minimize smoking exposure in children. Ultimately, promoting smoking cessation in parents could protect children from smoking exposure^47^.

On the other hand, in this study, the NNAL concentration was more correlated to having a father who smoked than having a mother who smoked. It is generally known that the more parents smoke, the higher their children’s SHS exposure^48^. Because fathers smoke more than mothers, their influence on children’s smoking exposure is more significant than that of mothers. In this study, the number of cigarettes smoked by fathers was higher than that smoked by mothers. In East Asian countries, which were influenced by Confucianism, men had a much higher smoking rate than women, and the number of cigarettes smoked was higher. Therefore, previous studies in South Korea and China have suggested fathers’ cessation of smoking as an intervention priority as a strategy to reduce their children’s exposure to secondhand smoke^49,50^.

Parents’ smoking cessation eliminates threats to their own and their children’s health, so relevant policies should be encouraged. To this end, increasing awareness of the harms associated with THS is required. The rate of smoking cessation attempts had increased following campaigns informing about the harms of SHS or THS, as parents believed quitting smoking would protect their children’s health^47,51^. Even if THS cannot be completely eliminated, awareness of the harms of SHS is likely to increase openness to legislation banning smoking at home^15^. Further, along with such shift in perception, implementing smoking cessation interventions can further boost the rate of successful smoking cessation^47^. Therefore, increasing the awareness of THS might be an effective strategy for inducing parents to quit smoking and, as a result, protecting children from tobacco exposure.

In a previous study based in Britain, which involved a population comparable to the population involved in the present study^33^. Children who lived with smoking parents had a higher saliva cotinine concentration even if they lived in a smoke-free home. This finding was consistent with our findings, suggesting that living with smoking parents might be a potent predictor of increased exposure to substances contained in tobacco. Importantly, as we controlled for smoking exposure in public places, which had not been considered in prior studies, our findings serve to strengthen the evidence presented by previous studies. In addition, by verifying the correlation with the parent’s NNAL concentration, the existing pieces of evidence that the parent’s smoking influences the concentration of children’s smoking-related biomarkers have been consolidated. Moreover, by using NNAL, which has a longer half-life, our results show those children are affected by exposure to tobacco substances over prolonged periods. Several previous studies had suggested that having a parent who smokes is the greatest predictor of SHS exposure in children^48^. However, this study can intuitively inform many smoker parents about the risk of smoking exposure. Therefore, from a public health perspective, our findings have important implications for the public. The indicator we used is a representative TSNA and a carcinogen, which chemically showed that carcinogens are detected in children’s bodies due to parental smoking. In conclusion, children of smoking parents are at a higher risk of SHS exposure, and even if parents strive not to smoke at home to prevent SHS, children are subject to short-term and long-term exposure to tobacco substances via THS or unrecognized SHS exposure.

This study has several limitations. First, children can give false responses that they deem more socially desirable^52^. In other words, children may falsely report that they do not smoke despite smoking. As a result, we cannot eliminate the possibility that some smoking children were classified as non-smokers in this study. Second, we cannot eliminate the likelihood of exposure to tobacco substances in places other than home. In this study, the NNAL concentration was higher in the older and boy groups. The smoking rate of adolescents increased with age and was higher among boys. Therefore, it suggested that boys are more likely to be exposed to smoking substances from their peers^53,54^. In addition, the possibility that few smokers belonged to the older and boy groups cannot be ruled out. Third, we asked about exposure to SHS in the past week in the survey, while NNAL has a half-life of 10–21 days^25^, which suggests that it might be more suitable to ask about exposure to SHS within the past month to reduce bias. To overcome these limitations, if data about chemical substances, such as surface nicotine and dust of NNK at home, could be collected in addition to biomarkers, exposure to SHS or THS at home could be more clearly assessed. Finally, e-cigarette users were not considered in our study because there are many non-responders for the question about e-cigarette smoking. Recently, the use of e-cigarettes has been rising among adults and adolescents, so subsequent studies should consider e-cigarettes when assessing SHS.

## Conclusions

We used tobacco-specific nitrosamines (TSNA), which were difficult to use in previous population studies; in addition, we controlled secondhand exposure in public places, one of the main biases in previous study. Prohibition of smoking at home is effective in preventing SHS exposure among children. However, it cannot completely prevent passive smoking. Parents who smoke expose their children to carcinogens. Even if parents strive not to smoke at home to prevent secondhand smoke, children are subject to short-term and long-term exposure to tobacco substances via thirdhand smoke and unrecognized secondhand smoke. To address this, there is a need to improve awareness regarding the recommended ways to protect children from smoking substances. However, the best way to protect children from toxic substances from smoking exposure is to quit smoking.
